# The Reconstruction of Nasal Cutaneous Defects With Locoregional Flaps

**DOI:** 10.7759/cureus.82841

**Published:** 2025-04-23

**Authors:** Lentiona Basiari, Maria C Michali, Ioannis D Komnos, Alexandros Georgolios, Victoria Tsoumani, Magdalini Bizoglou, Dimitra G Simou, Georgios V Psychogios

**Affiliations:** 1 Department of Otorhinolaryngology, Head and Neck Surgery, University Hospital of Ioannina, Ioannina, GRC; 2 Department of Otolaryngology, Head and Neck Surgery, University hospital of Ioannina, Ioannina, GRC; 3 Department of Otolaryngology, Head and Neck Surgery, University Hospital of Ioannina, Ioannina, GRC

**Keywords:** aesthetic subunit, cheek, external nose, glabella, local flap reconstruction, nose anatomy, regional flap

## Abstract

The nose occupies the central position on the face and may be the most difficult facial feature to reconstruct due to its complex anatomy and function. Cancer resection and trauma are the most common causes of nasal defects. The goal in such cases is to select the most appropriate option for a given defect to preserve nasal function and to achieve the best aesthetic outcome. The most important aspect of a successful reconstruction is preoperative planning. Although small defects may only require a single procedure and primary closure might be an option, multiple procedures are typically needed to reconstruct a more extensive defect to prevent nasal distortion or collapse. Locoregional flaps are the first choice for reconstruction as they use nearby tissue, providing an excellent color and texture match with the best results. The objective of this article is to engage in a review of the most common locoregional flaps used to reconstruct cutaneous nasal defects. We analyze the most commonly used locoregional flaps, as well as their advantages and disadvantages.

## Introduction and background

The art of nasal reconstruction dates back many years to the ancient period in India, where the brutal practice of nasal amputation that existed as a form of punishment prompted the search for fixing the organ, marking the birth of plastic surgery. Sushruta, known as the Father of Plastic Surgery, was the first to describe nasal reconstruction in 600 BC, by using a cheek flap and a leaf template [1]. The nose is located centrally on the face and has an important aesthetic and functional role related to breathing and smelling. Hence, nose reconstruction should be performed with extreme caution. Recent research has shown that patients with nasal cutaneous deformities tend to have difficulties interacting with others in social situations, negatively impacting their quality of life [2]. In addition, deformities in the area of the nasal valve have a negative impact on nasal breathing, which is related to a worse quality of life.

The most common cause of nasal cutaneous defects is nonmelanoma skin cancer, such as basal cell carcinoma (BCC) and squamous cell carcinoma (SCC) [3-5]. The main goal in these cases is to achieve excision of the lesion without any residual tumor cells in the margin. This margin-free resection of these cutaneous malignancies can lead to significant defects that must be carefully reconstructed. Preoperative planning is crucial to obtain optimal results. Determining the size and location of the defect and selecting the most suitable donor site are key factors in achieving the best aesthetic and functional result while reconstructing complex nasal defects. A thorough knowledge of the superficial anatomy of the nose and its aesthetic subunits is critical in decision-making. Surgeons should carefully analyze the defect preoperatively and determine which layers of the nose are missing and must be replaced. Local flaps, defined as flaps that are next to the defect, and regional flaps, defined as flaps located in the same region as the defect, are excellent options for reconstructing nasal cutaneous defects. In this article, via an extensive review of the literature, we aim to provide a detailed assessment of the most common locoregional flaps used for nasal reconstruction, depending on the location of the defect, which we think would be very helpful, especially for new or trainee surgeons.

## Review

Methods

An extensive review of the literature was conducted on the PubMed and Google Scholar databases using the following keywords: “nasal reconstruction”, “nasal aesthetic subunit”, “full thickness defect”, “local nasal flap”, “nasal tip reconstruction”, “nasal ala reconstruction”, “dorsal nasal flap”, “glabellar flap”, “forehead flap”, “nasolabial flap”, “bilobed flap”, “cheek flap”. Articles about free flaps, skin grafts, or other alternative methods for nasal reconstruction were excluded. Fifty-three articles containing information about locoregional flaps were selected and studied. The most common locoregional flaps were studied in detail regarding their advantages and disadvantages, indications, and the parameters of their designation.

Surgical anatomy of the external nasal pyramid

The anatomic components of the external nose include the skin, the soft tissue envelope, and the osseocartilaginous framework [6]. Skin characteristics are very important during preoperative planning, and their qualities vary in different nasal regions. The thickness of the skin differs throughout the surface of the nose. According to Lessard and Daniel, the skin is thicker in the caudal part of the nose, with an average thickness of approximately 1.25 mm, and thinner (0.6 mm) in the upper half of the nose at the rhinion [7]. The skin also becomes thinner, progressing from the tip to the columella and alar margin [8]. The caudal part of the nose is richer in sebaceous glands compared to the upper half, and the skin is oilier. As we progress from the tip to the rhinion, the skin tends to become more mobile and less adherent to the subcutaneous tissue [6,8,9]. It is important to keep in mind that relaxed skin tension lines run horizontally throughout the nose surface except for the ala of the nose, where their orientation is oblique [10]. Placement of incisions parallel to these lines is recommended to avoid wound tension and achieve an acceptable scar.

The soft tissue envelope, which is located between the skin and the osseocartilaginous framework, is made up of four layers: 1. the superficial fatty layer, which is connected to the dermis; 2. the fibromuscular layer known as the nasal subcutaneous muscular aponeurotic system (SMAS) which contains the mimetic muscles; 3. the deep fatty layer that contains the blood vessels and nerves that supply the external nose; 4. the perichondrium and periosteum [8,9,11,12]. The avascular plane of dissection that is used during rhinoplasty is located between the deep fatty layer and the perichondrium and periosteum. The blood supply of the external nose derives from branches of the external carotid artery through the facial artery and internal maxillary artery, and branches of the internal carotid artery through the ophthalmic artery [9]. These branches provide a rich blood supply for the designation of random and axial locoregional flaps.

The bony component of the osteocartilaginous framework is composed of the paired nasal bones, which articulate cranially with the nasal process of the frontal bone, laterally with the ascending process of the maxilla, and caudally with the upper lateral cartilages. The nasal bones are thicker cranially, and they articulate with the perpendicular plate of the ethmoid bone. The area between the nasal bones, the perpendicular plate of the ethmoid bone, the upper lateral cartilages, and the nasal septum is known as the keystone area and is of crucial importance for the support of the nose [6]. The cartilaginous nasal skeleton contains the upper lateral cartilages, the lower lateral cartilages, and the sesamoid and accessory cartilages. The external nasal valve is composed of the nasal ala and the supporting structures that include the columella and the lateral crura of the lower lateral cartilage [6].

Aesthetic subunits and clinical significance

The concept of the aesthetic subunits of the nose was first proposed by Burget and Menick in 1985 [13]. They divided the nose topographically into the following aesthetic subunits: the tip, dorsum, columella, paired ala, sidewalls, and soft triangles. According to them, if more than 50% of the subunit is missing after the resection of the lesion, then the reconstruction should include the entire aesthetic subunit to achieve better cosmetic results. The subunits differ in skin characteristics (color, thickness, and amount of sebaceous glands) and underlying skeletal support. The underlying skeletal framework defines the transition lines and the natural boundaries between the subunits [14]. The domal part of the lower lateral cartilage defines the subunit of the tip, which transits to the dorsum at the level of the supratip area. The upper lateral cartilage and the articulation of the ascending process of the maxilla with the nasal bone determine the border between the dorsum and the sidewall subunit. The upper lateral cartilages also have a very important functional role as part of the internal nasal valve. The alar crease is defined by the lateral crura of the lower lateral cartilage and is the region where the sidewall transits to the ala unit, which is supported mainly by fibrofatty tissue. Meticulous reconstruction of the ala is important because defects of this subunit can result in alar collapse and dysfunction of the external nasal valve, impacting airflow and patients’ quality of life [12]. The soft triangle unit is placed between the columella and the ala and is prone to irregularities due to scarring when it is violated by intranasal incisions [8].

Reconstructions that follow the principle of the aesthetic subunit tend to have better cosmetic results because incisions and scars placed on the natural borders of the subunits can be better camouflaged. Although respecting the nasal aesthetic subunit principle is important, surgeons should also evaluate factors like age, other medical comorbidities, tobacco use, and anesthesia-related complications when planning reconstruction.

Description of the most common locoregional flaps for nasal reconstruction

Nasolabial Flap

The nasolabial flap was first described by the German surgeon Johann Friedrich Dieffenbach. It is a random flap that depends on the rich subcutaneous vascular plexus of the nasolabial region that derives from branches of the facial artery, angular artery, and infraorbital artery [15]. This flap utilizes the mobile and abundant skin of the medial cheek and nasolabial region mainly for the reconstruction of defects of the ala and caudal part of the nasal sidewall. The skin of the nasolabial region matches the color and texture of the skin in these areas of the nose, making it a suitable option for reconstruction [16]. Also, due to the robust vascularity, this flap can support free cartilage grafts in cases of complex alar reconstruction [17]. Donor site morbidity is minimal, and the scar is camouflaged in the nasolabial fold.

**Figure 1:**
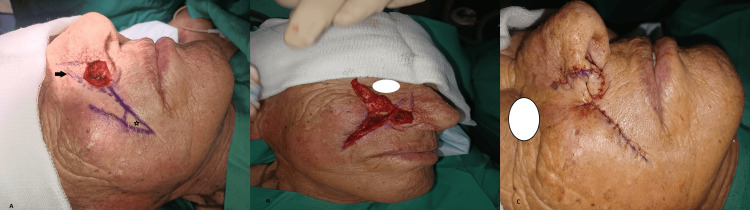
Transposition nasolabial flap A) Intraoperative designation of a transposition nasolabial flap for the reconstruction of a 1.5 cm defect of the ala after resection of basal cell carcinoma. A Burow’s triangle adjacent to the defect (black arrow) and another one from the flap (asterisk) are excised. B) Elevation of the flap. C) Closure of the defect and donor site. The donor site incision is placed in the nasolabial crease (photos from Dr. Basiari's archive)

Different types of nasolabial flaps have been described in the literature. It can be used as a transpositional flap or as an interpolated flap. When used as a transpositional flap (Figures 1A-1C), it is performed as a one-stage procedure, and the base of the flap is adjacent to the defect. Usually, the base of the flap is superiorly placed, allowing maximal pedicle movement. A disadvantage of the transpositional nasolabial flap can be the obliteration and distortion of the alar-facial sulcus [16-18]. In the case of an interpolated nasolabial flap, it can be designed as an interpolated subcutaneous pedicle flap or as an interpolated cutaneous pedicle flap. The reconstruction, in this case, requires two stages. In the second stage, division of the pedicle is performed, usually three weeks after flap elevation. Interpolated nasolabial flaps do not distort the alar-facial groove. When designed with a subcutaneous pedicle, a wider dissection is required, posing a risk of injury to the facial nerve branches [15].

The nasolabial flap might also be used for the reconstruction of the columella. In this case, the flap is folded, and a cartilaginous strut graft is included. Lastly, in cases of full-thickness defects when other options, such as septal or buccal flaps, are not available, the nasolabial flap can be used for nasal lining reconstruction [15-17].

Bilobed Flap

**Figure 2:**
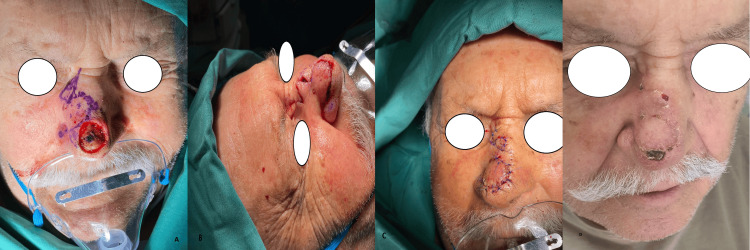
The bilobed flap A) Reconstruction of a 1.7 cm defect of the nasal tip with a laterally based bilobed flap. The designation of the two lobes of the flap is shown in the figure. B-C) The first lobe of the flap is used to close the defect, the second lobe is used to close the donor site of the first lobe, and primary closure is performed for the donor site of the second lobe. D) Postoperative view of the patient after two weeks (photos from Dr. Basiari's archive)

The bilobed flap (Figures 2A-2D) was first described by Esser, and in 1989, it was modified by Zitelli, who proposed smaller transposition arcs for the flap [19]. It is a random pattern double transposition flap which is used especially for the reconstruction of defects in the lower third of the nose. This flap is an ideal choice for defects involving the nasal tip, supratip, or near the tip area with a maximum diameter of 1.5 cm [16,20,21]. It has also been used successfully for the reconstruction of small (< 1cm) defects of the alar rim and large defects of the ala [21-23]. The primary lobe has an equal size to the defect and is used for defect closure. The secondary lobe usually has a size of two-thirds of the primary lobe and is designed to close the donor site of the primary lobe. Primary closure is used for the secondary lobe donor site [18]. The angle of transposition of the lobes is usually 45⁰, and the elevation of the flap is placed above the perichondrium and the periosteum, including the SMAS in the flap [24,25]. A wide undermining of the flap, the defect, and the donor site at this level is important to avoid distortion and pin cushioning deformities [16]. The bilobed flap for nasal tip and alar defects is usually designed as a laterally based flap to obtain better cosmetic results, positioning the standing cutaneous deformity within or parallel to the alar groove [16,18,26]. However, medially based flaps have also been used successfully in the literature [23]. In this case, there is a small risk of distortion and deformity of the alar groove mimicking a dorsal hump [25].

For defects of the lower third of the nose that involve the entire depth of the subcutaneous tissue, the bilobed flap is more suitable compared to skin grafts, which can lead to visible nose depression [26]. Finally, surgeons must keep in mind that the bilobed flap does not follow the principle of the aesthetic subunits, and incisions are not placed in natural boundaries. Despite this, when meticulous closure in layers is performed, aesthetic results are quite acceptable [25,26].

Rintala Flap

**Figure 3:**
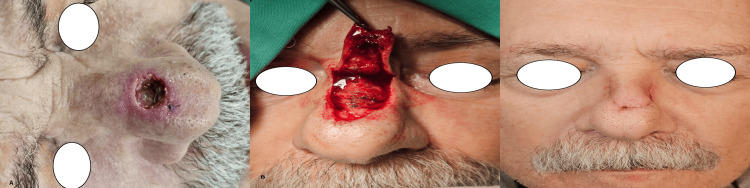
Rintala flap A) A 1 cm defect located between the dorsum and the tip after the resection of a basal cell carcinoma. A Rintala flap was chosen for the reconstruction. B) Harvesting the flap. C) Postoperative view after 10 days (photos from Dr. Basiari's archive)

The Rintala flap (Figures 3A-3C) was first described in 1969 [27] as a random pattern dorsal nasal advancement flap performed in one stage. It is designed as a rectangular superiorly based flap, which is used for the reconstruction of midline defects located in the area from the nasal root to the tip [28-30]. Two parallel incisions are placed bilaterally along the nasal sidewall, extending from the corner of the defect to the medial canthal level or suprabrow level, depending on the desired length. Usually, at the base of the flap, a Burow triangle is excised on both sides. When placed at the suprabrow level, Burow’s triangles provide a more extended flap [28,29]. The flap is raised from its distal part in a supraperiosteal plane, and it provides excellent aesthetic results due to color match, adequate thickness, low ischemia risk, and scars that are placed in areas of natural shadow [30]. Repair in two layers, subcutaneous and skin, is necessary to avoid pincushion deformities. The Rintala flap is a good alternative for the reconstruction of the nasal tip, supratip, and midline dorsal nasal defects up to 2 cm. In combination with other local flaps like the cheek advancement flap, it can be used to close large defects of the tip and dorsum up to 4 cm [28].

Dorsal Nasal Flap

**Figure 4:**
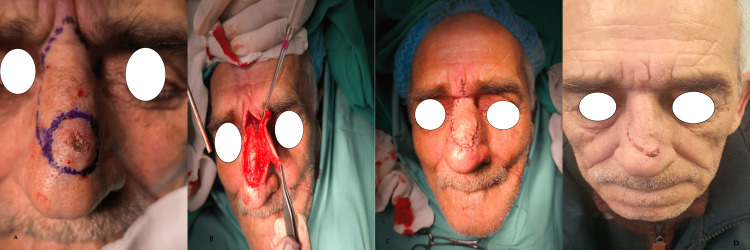
Dorsal nasal flap A) A patient with a basal cell carcinoma of the supratip area. Designation of the excision of a dorsal nasal flap for reconstruction. An inverted V incision is placed in the glabellar region, and a vertical incision is drawn until the defect. B) Elevation of the flap and rotation. C) Closure of the defect of the donor site. D) Patient view three weeks postoperatively (photos from Dr. Basiari's archive)

The dorsal nasal flap (Figures 4A-4D) was first described by Rieger as a random pattern rotational advancement flap with a wide skin pedicle located on the lateral side of the nose and a Z-plasty closure of the glabellar defect for the reconstruction of moderate-size defects of the lower third of the nose in one stage [31]. Later, Marchac and Toth [32] described a modified version of the Rieger flap, designed as an axial flap based on a branch from the angular artery, which allowed a greater degree of flap rotation and a V-Y closure of the glabellar defect. The indications of the original flap include mainly reconstruction of defects of the lower half of the nose, especially those of the nasal tip, with a size up to 2 cm in diameter and with a distance of at least 5 mm from the alar rim [33-36]. For the designation of the flap, an inverted V incision is usually placed in the glabella and is continued either ipsilaterally or contralaterally along the sidewall-cheek junction to the superior side of the defect, mobilizing abundant skin from the glabellar and dorsal area of the nose and camouflaging the incision in areas of natural shadow [17]. The elevation of the flap is performed in a submuscular plane on the dorsum area and is transferred in a subcutaneous plane in the glabellar region [14].

Many modifications of the original flap have been described in the literature by many authors, who have reported the benefits of the proposed modifications in different cases. In 1973, Rigg proposed the concept of the heminasal flap using only part of the skin of the nasal dorsum, which provides an adequate option for smaller defects. In this case, the lateral incision is extended from the glabella to the defect along the junction of the dorsum-sidewall aesthetic subunits [33]. In 1995, Green and Angeles proposed a new version of the dorsal nasal flap with an ipsilateral pedicle designed to avoid the extension of the flap into the glabellar region and prevent the transfer of the thick glabellar skin to the region of the medial canthus, which is typically characterized by thin skin [33,37].

In 2007, Bitgood and Hybarger described the use of a modified dorsal nasal flap technique for the reconstruction of larger size defects up to 35 x 40 mm in patients that are not good candidates for paramedian forehead flap, such as elderly patients who cannot afford to undergo multistage procedures, patients that due to socioeconomic status cannot afford long time apart from work and patients that do not consent for a forehead flap due to their dilemma regarding flap appearance until revision [35]. Eren and Beden also described the reconstruction of larger than 3 cm defects in four patients with dorsal nasal flaps, which had good aesthetic results, as well as minor complications such as minor wound dehiscence and asymmetric nasal tip elevation [36]. Redondo et al. [38] proposed an elongated dorsal nasal flap to reconstruct large defects, while Wentzell [39] proved that full-thickness defects can be reconstructed with a dorsal nasal flap.

In conclusion, the dorsal nasal flap and its modifications provide an excellent choice for one-stage reconstruction of variable-size defects of the lower half of the nose. This flap provides adequate skin color, thickness, and texture, especially for the reconstruction of the nasal tip area.

Paramedian Forehead Flap

The paramedian forehead flap is one of the most used flaps for the reconstruction of large, complex nasal defects involving one or multiple subunits. It is designed as an axial pattern interpolated flap based on the supratrochlear artery and is usually performed in a two-stage procedure. Since its first description in the ancient Sanskrit text by Sushruta Samhita, the flap technique has evolved and undergone modifications [1,40]. It was initially designed as a median forehead flap involving bilateral supratrochlear arteries in the pedicle, which limited its length. Millard was the first to prove that it was not necessary for flap viability to include both supratrochlear arteries and proposed the transposition of forehead tissue based on a unilateral paramedian blood supply, narrowing the pedicle, and increasing the length of the flap [41]. This flap remains the most appropriate choice for the reconstruction of total and subtotal defects of the nose.

Many anatomical studies have demonstrated the presence of a rich anastomotic plexus deriving from branches of the supratrochlear, supraorbital, and dorsal nasal artery centered on the medial canthal area, which is responsible for the blood supply to the forehead region [40,41]. The pedicle has a width of 15 mm and is based on the supratrochlear artery, which is usually located 1.7-2.2 cm from the midline [17,41-45]. A Doppler ultrasound is used by many surgeons for a more accurate location of the supratrochlear artery, especially if a narrower pedicle of up to 10 mm is needed for optimal flap designation [17,46]. The supratrochlear artery exits from the orbit and travels in a plane superficial to the corrugator muscle and deep to the orbicularis oculi [44]. The artery traverses the frontalis muscle approximately 2 cm above the orbital rim and continues in a subcutaneous plane [17,44].

In some cases, to achieve maximal flap rotation, the subcutaneous portion of the flap is removed, and a split-thickness skin graft is used to protect the vascular pedicle [17]. The flap is harvested in a subgaleal plane in the forehead area and continues subperiosteally proximally to avoid damage to the supratrochlear artery [14,17,41,47]. The donor site is usually closed primarily, but it cannot be closed entirely in cases of large defects and is left to heal with secondary intention. Thinning of the distal part of the flap should be performed carefully, especially in smokers [14]. The paramedian forehead flap is usually performed as a two-stage procedure. During the first stage, elevation and transfer of the flap are performed, while during the second stage, which takes place after three weeks, pedicle division is accomplished [48]. In the literature, a three-staged procedure and a one-staged procedure of the paramedian forehead flap have also been described [48,49].

In conclusion, the paramedian forehead flap remains a workhorse of nasal reconstruction with few contraindications, especially in elderly patients with comorbidities.

Cheek Advancement Flap

**Figure 5:**
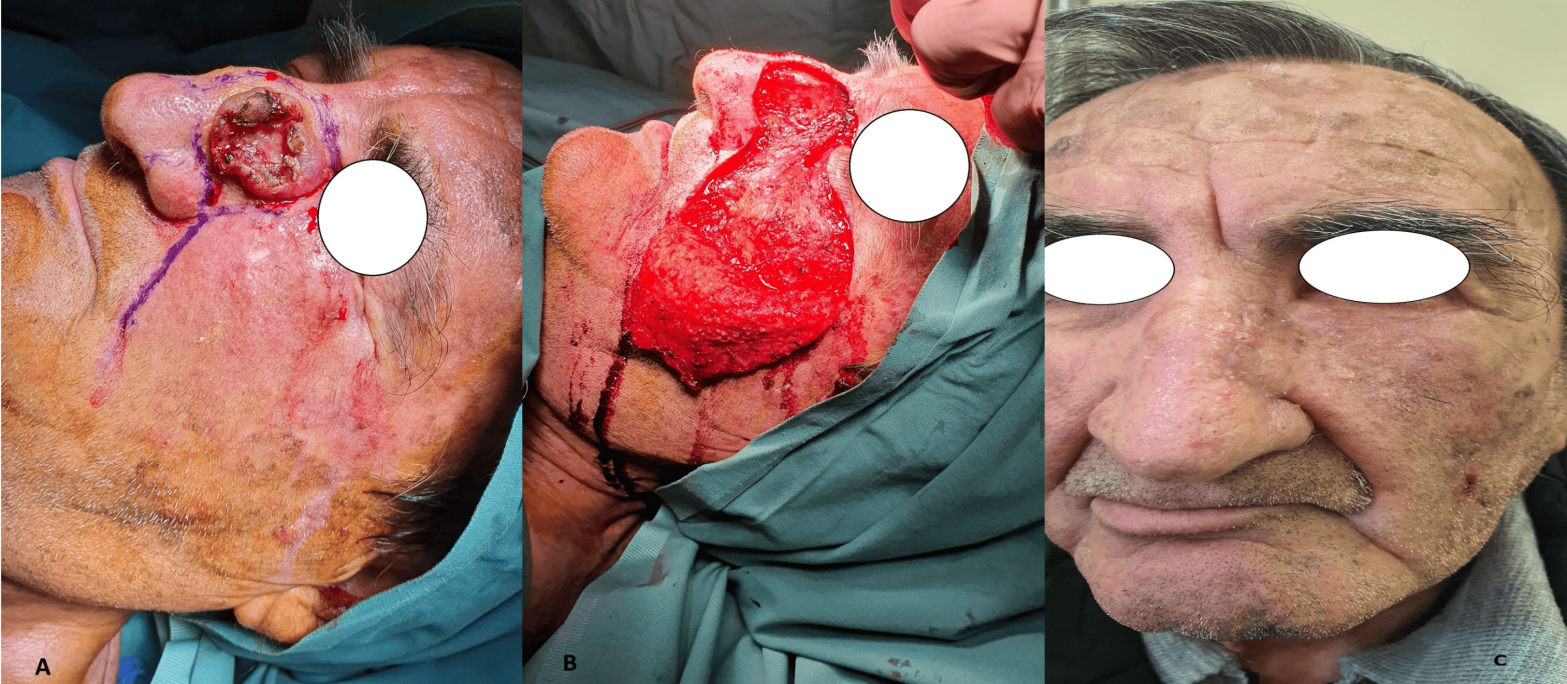
Cheek advancement flap A) A 75-year-old male patient with a large squamous cell carcinoma involving the dorsum and the sidewall unit. After resection, a defect of 3 cm was reconstructed with a cheek advancement flap. B) Elevation of the flap superficial to the SMAS. C) Postoperative view after 2 months with optimal cosmetic results and no signs of ectropion (photos from Dr. Basiari's archive) SMAS: subcutaneous muscular aponeurotic system

Reconstruction of larger defects of the lateral nose involving the medial region of the cheek is challenging. In these cases, the cheek advancement flap provides an optimal choice because it offers an ideal tissue match in the context of skin color, thickness, and texture. Especially large defects of the nasal sidewall and dorsum can be reconstructed with this flap with optimal cosmetic results (Figure 5A-5C). The flap is designed with the inferior incision placed in the nasolabial crease and the superior horizontal incision placed in a natural lower eyelid crease, extending toward the ear [18]. Care should be taken to avoid postoperative ectropion. Elevation of the flap takes place superficial to the SMAS [18,50]. Partial necroses of the distal part of the flap might occur in cases of an extended cheek flap [51].

 *Glabellar Flap*

The glabellar flap has usually been used as a V-Y advancement flap with a random pattern blood supply for the reconstruction of defects located in the upper third of the nose [52]. Numerous modifications of the glabellar flap have been proposed by many surgeons in the literature. It has been used as a rhomboid transposition flap [18] or as a rotation advancement flap [52]. Field [53] described a transposition glabellar flap, which he called the “banner” flap, for the reconstruction of small to moderate-sized defects of the proximal nose. Disadvantages of the glabellar flap may include asymmetry of the interbrow region and the thicker skin of the glabella in contrast to the thin skin of the recipient site [52].

## Conclusions

Nasal reconstruction of cutaneous defects often poses a challenge for the surgeon. The primary goal of resection-free margins often leads to significant defects. For the reconstruction of these defects with optimal esthetic and functional results, it is critical to have a thorough knowledge of the complex anatomy and the characteristics of the esthetic subunits of the nose. Detailed preoperative planning is crucial. Factors including patient age, comorbidities, anticoagulant therapy, and smoking status should be evaluated in the process of decision-making. A variety of locoregional flaps are available for the surgeon, and the optimum choice should be selected and personalized for each patient.
